# Atopic asthmatic immune phenotypes associated with airway microbiota and airway obstruction

**DOI:** 10.1371/journal.pone.0184566

**Published:** 2017-10-20

**Authors:** Benjamin A. Turturice, Halvor S. McGee, Brian Oliver, Melissa Baraket, Brian T. Nguyen, Christian Ascoli, Ravi Ranjan, Asha Rani, David L. Perkins, Patricia W. Finn

**Affiliations:** 1 Department of Medicine, Division of Pulmonary, Critical Care, Sleep, and Allergy, University of Illinois at Chicago, Chicago, IL, United States of America; 2 Department of Microbiology and Immunology, University of Illinois at Chicago, Chicago, IL, United States of America; 3 Respiratory Cellular and Molecular Biology, Woolcock Institute of Medical Research, Sydney, NSW, Australia; 4 Molecular Biosciences, School of Life Sciences, University of Technology Sydney, Sydney, NSW, Australia; 5 South Western Sydney Clinical School, University of New South Wales, Liverpool, NSW, Australia; 6 Department of Respiratory Medicine and Sleep Medicine and Ingham Institute Applied Medical Research, Liverpool Hospital, Liverpool, NSW, Australia; 7 Department of Medicine, Division of Nephrology, University of Illinois at Chicago, Chicago, IL, United States of America; 8 Department of Surgery, University of Illinois at Chicago, Chicago, IL, United States of America; 9 Department of Bioengineering, University of Illinois at Chicago, Chicago, IL, United States of America; Imperial College London, UNITED KINGDOM

## Abstract

**Background:**

Differences in asthma severity may be related to inflammation in the airways. The lower airway microbiota has been associated with clinical features such as airway obstruction, symptom control, and response to corticosteroids.

**Objective:**

To assess the relationship between local airway inflammation, severity of disease, and the lower airway microbiota in atopic asthmatics.

**Methods:**

A cohort of young adult, atopic asthmatics with intermittent or mild/moderate persistent symptoms (n = 13) were assessed via bronchoscopy, lavage, and spirometry. These individuals were compared to age matched non-asthmatic controls (n = 6) and to themselves after six weeks of treatment with fluticasone propionate (FP). Inflammation of the airways was assessed via a cytokine and chemokine panel. Lower airway microbiota composition was determined by metagenomic shotgun sequencing.

**Results:**

Unsupervised clustering of cytokines and chemokines prior to treatment with FP identified two asthmatic phenotypes (AP), termed AP1 and AP2, with distinct bronchoalveolar lavage inflammatory profiles. AP2 was associated with more obstruction, compared to AP1. After treatment with FP reduced MIP-1β and TNF-α and increased IL-2 was observed. A module of highly correlated cytokines that include MIP-1β and TNF-α was identified that negatively correlated with pulmonary function. Independently, IL-2 was positively correlated with pulmonary function. The airway microbiome composition correlated with asthmatic phenotypes. AP2, prior to FP treatment, was enriched with *Streptococcus pneumoniae*. Unique associations between IL-2 or the cytokine module and the microbiota composition of the airways were observed in asthmatics subjects prior to treatment but not after or in controls.

**Conclusion:**

The underlying inflammation in atopic asthma is related to the composition of microbiota and is associated with severity of airway obstruction. Treatment with inhaled corticosteroids was associated with changes in the airway inflammatory response to microbiota.

## Introduction

Asthma is a heterogeneous disease. Asthma phenotypes have been established on the basis of underlying inflammation that may be predictive of treatment response and severity of disease. For example, atopic asthma has been associated with T-helper (Th) 2 responses and development of symptoms early in life, whereas other phenotypes have been associated with non-Th2 inflammation and development of symptoms later in life [1–3]. Recently, additional T-cell subsets have been determined to have a role in the pathogenesis of atopic asthma, such as Th17 and T-regulatory (Treg) cells [4–9]. These subsets are responsible for immunity to specific microbes or regulation of inflammation.

The composition of the airway microbiome in asthmatics has been associated with clinical features, such as symptom control, severity of airway obstruction, and response to corticosteroid therapy [10–13]. Pathogenic bacteria (e.g., Haemophilus, Streptococcus, Moraxella, Klebsiella, and Rickettsia) in the upper and lower airways have been associated with the diagnoses of asthma and potentially hold prognostic value for future diagnosis of asthma. [12, 14–16]. Bacteria of the upper airway and oral cavity can also be identified within the lower airways. Notably, a high abundance of supraglottic taxa in the lower airways of healthy individuals has been associated with Th17-like cytokine profiles in bronchoalveolar lavage (BAL) along with increased lymphocyte and neutrophil counts in BAL [17, 18]. Thus, there is a strong link with the airway microbiome and local airway inflammation, specifically with respect to T-cell phenotypes, in healthy individuals.

Recently, the relationship between the microbiome of the airways and immune response in the airway of asthmatics has been explored [12, 19, 20]. Prior studies focused on changes in the airway microbiome related to the presence of Th2 or Th17 responses in bronchial epithelium and the cellularity of eosinophils and neutrophils in the airways. We hypothesized that there are unique associations between inflammation, airway microbiome, and pulmonary function in asthmatics. We analyzed cytokine concentrations in bronchoalveolar lavage of young adult, atopic asthmatics (n = 13) and their relationship to the airway microbiome and lung function. In addition, we tested the impact of a short term, commonly used asthma medication, fluticasone propionate (FP) on airway inflammation and microbiome. Asthmatics were compared to age matched non-asthmatic individuals (n = 6) and to themselves after treatment with fluticasone propionate. The subjects were free of smoking, signs of respiratory infection, and corticosteroid therapy in the preceding months to control for potential confounders that can affect the microbiome and inflammation [11, 21, 22]. We identified two unique cytokine profiles that were associated with airway obstruction and compositional changes in the microbiome. These inflammatory changes were reduced after FP therapy, as well as association between the cytokines and the microbiome composition. This indicates that the relationship between inflammation and the microbiome is associated with pulmonary function in asthmatics.

## Materials and methods

### Subjects

The study was approved by the Ethics Review Committee of Royal Prince Alfred Hospital in the Sydney South West Area Health Service, protocol number X02-0137. Written informed consent was obtained from all subjects, and no minors were included. BAL samples were transferred and processed under our University of Illinois at Chicago approved IRB protocol number 2015–0085.

Method and subject recruitment for bronchoalveolar lavage, allergen testing, serum IgE, exhaled nitric oxide (NO), and spirometry has been described previously [23]. Briefly, bronchoscopy took place following fasting for the previous 12 hours. Subjects were sedated with doses of midazolam and alfentanil, based upon the subject’s initial response to treatment. Topical anaesthesia (lidocaine) was introduced through the fiberoptic bronchoscope, with the maximum dose determined by the subject’s weight. Following wedging of the bronchoscope in the sub segmental airways, 4 x 60 ml (i.e. 240ml) aliquots of warmed sterile sodium chloride (0.9% W/V) were instilled, with suctioning after each, into a single, sterile, ice-cold glass reservoir. Pooled bronchoalveolar (BAL) fluid was placed on ice and transported to the laboratory for processing. BAL was centrifuged at 500 x g for 10, and BAL fluid aspirated and stored at -80 until use. Adult volunteers between the ages of 19–30 years were recruited to participate in the study. Asthmatic volunteers with intermittent or mild persistent or moderate persistent atopic asthma (according to Global Initiative for Asthma, Global Strategy for Asthma Management and Prevention, NIH Publication, Updated 2005) were included if they met the following inclusion criteria: asthma symptoms in the preceding 12 months and positive mannitol bronchial provocation test. Individuals were excluded from the study if they had baseline FEV1 less than 60% predicted, inhaled or systemic corticosteroid treatment in the preceding 2 months, smoking in the preceding 12 months, greater than 5 pack year smoking history and symptoms of upper respiratory tract infection in the past 6 weeks.

### Study design

Subjects were initially screened at a baseline visit for their medical history, physical examination, exhaled NO, spirometry, mannitol challenge, allergen skin test, and Juniper Asthma Control Questionaire Score. One to four weeks post-intial visit subjects underwent bronchodilator challenge, blood draw for serum IgE, and bronchoscopy with lavage and biopsy. As part of the original Baraket et al. study [23], asthmatic subjects were then randomized into two dosing strategies of inhaled fluticasone propionate (100 μg/BID and 500 μg/BID) delivered by metered dose aerosol and large volume spacer. Subjects were provided with salbutamol metered dose aerosol (100 μg) to be used as needed for asthmatic symptoms. Subjects were on no other medications during the study period. Asthmatic subjects were assessed six weeks after initiating of inhaled fluticasone propionate for exhaled NO, spirometry, mannitol challenge, and Juniper Asthma Control Questionaire Score. Seven weeks after initiating inhaled fluticasone propionate subjects underwent bronchodilator challenge, and bronchoscopy with lavage and biopsy.

### Spirometry and lung function reference values

Spirometric measurements were performed with a Vmax spirometer (SensorMedics, Yorba Linda, California) using the closed circuit technique. Subjects were asked to withhold short acting bronchodilators for at least 8 hours prior to the visit. No subjects were taking long acting beta-2 agonists. Subjects were asked to not to consume caffeine or engage in vigorous exercise on the morning of the visit. Predicted values were determined based on race, height, age, and sex using the reference equations derived from NHANES III. Spirometry values were interpreted using the ATS/ERS guidelines [24, 25].

### Multiplex cytokine and chemokine assay

100 μL of BAL was centrifuged at 10,000 x g for 10 min to clear any cellular debris. Acellular BAL was assessed for concentrations of sCD40L, MCP-1, MIP-1β, TNF-α, G-CSF, GM-CSF, IFN-γ, IL-1β, -2, -4, -5, -6, -7, -8, -10, -12(p70), -13, -17A, -17F, -21, -22, -23, -25, -31, and -33 using Bio-Plex Pro™ Human Th17 Cytokine Panel 15-plex and Human Cytokine 17-plex assays (Bio-Rad). Concentrations were determined using the Bio-Plex® 200 system and software. IL-5 was excluded from analysis as no samples had concentrations within the detectable range. Samples outside detectable limits were given the concentration of zero.

### DNA extraction, metagenomic DNA library construction, and sequencing

BAL was removed of eukaryotic cells by centrifugation at 1000 x g for 10 min. To extract microbial DNA from BAL, 1 mL of BAL was centrifuged at 22,000 x g for 2 hours at 4°C. Supernatants were removed and the pellet was treated with Lysozyme (Fisher Bio) in AL Buffer (Qiagen) to lyse microbial cells. DNA was extracted from lysed pellets using QIAamp Virus Spin Minelute kit (Qiagen) per manufacturer’s protocol. DNA libraries for sequencing were constructed using Nextera XT DNA Library Prep Kit (Illumina). The quality and quantity of all the DNA libraries were analyzed with DNA High Sensitivity Kit (Agilent) on the 2100 Bioanalyzer Instrument (Agilent) and Qubit (Invitrogen). The samples were sequenced as described previously [26]. DNA libraries were pooled in equimolar concentration and were sequenced following manufacturer’s protocol by multiplexing on Illumina MiSeq using the v3-600 kit for 301 paired-end read length and included an additional 6 cycles for the index. The average sequencing depth was 5,065,879 reads per sample.

### De novo genomic assembly and annotation of metagenomic sequences

FASTQ files for each sample were mapped against the human genome at a fraction size of 0.500 bp and 95 percent identity to remove contaminating human genomic DNA. There was an average of 116,200 unmapped reads (i.e. non-human or microbial reads) and 18,900,000 base pairs per sample. Unmapped reads were assembled into contiguous sequences at minimum 300 base pair using CLC Genomics Workbench (CLC Bio). The assembly files were uploaded on to the public database server MG-RAST [27] and taxonomy was assigned with the minimum parameters of 60% identity, 10^−5^ e-value, and 60 amino acids alignment length using MG-RAST’s M5NR database. Data for the samples that were sequenced have been uploaded onto MG-RAST and are available under the Project ID: 12564.

### Statistical analysis

All statistical analyses were performed in *R* (Version 3.3) unless otherwise specified. Cytokines were standardized using *z-*score and principal component analysis (PCA) was performed using Euclidean distances. Differential centroid testing for PCA was performed by permutation ANOVA (PERMANOVA) using *Adonis*. For determination of asthmatic phenotypes, unsupervised clustering of the first six principal components was used for hierarchical clustering using Pearson’s correlations as distances and Unweighted Pair Group Method with Arithmetic Mean (UPGMA) as the clustering algorithm. Optimal cluster number was assessed using average silhouette width and cluster stability was determined using consensus clustering [28]. Cytokine and chemokine concentration differences were tested using *limma* and pair-wise contrasts [29]. Linear regression and ordinal logistic regression for cytokines or modules was performed using univariate or multiple predictors. For multiple adjustment, modules were adjusted for treatment with FP and the interaction of the module with treatment. Modules of cytokines were identified using significant Pearson’s correlations controlled for multiple hypothesis testing (*P* < 0.05, *Q* < 0.05) and clustered using complete-linkage algorithm. Clusters were said to be modules if five or more cytokines had interactions with each other to control for clusters composed of spurious interactions. Detrended correspondence analysis and microbiome correlation with phenotypes and clinical variables was performed using *vegan*. Differential abundance of specific species was tested by pair-wise contrasts using *DESeq2* [30]. Slopes of univariate linear regressions with DCA1 as a linear predictor of cytokines or modules were compared using ANCOVA. All subject’s clinical data was tested for significant differences via Student’s T-test for continuous data or Chi-squared analysis with bootstrap approximation for distribution for categorical data. *P* < 0.05 was considered statistically significant. For multiple testing procedures, *Q* values were determined to control for Type I error [31]. *P* < 0.05 and *Q* < 0.1 was considered significant for multiple testing procedures.

## Results

### Unsupervised clustering of cytokines and chemokines identifies asthmatic phenotypes

**Fig 1:**
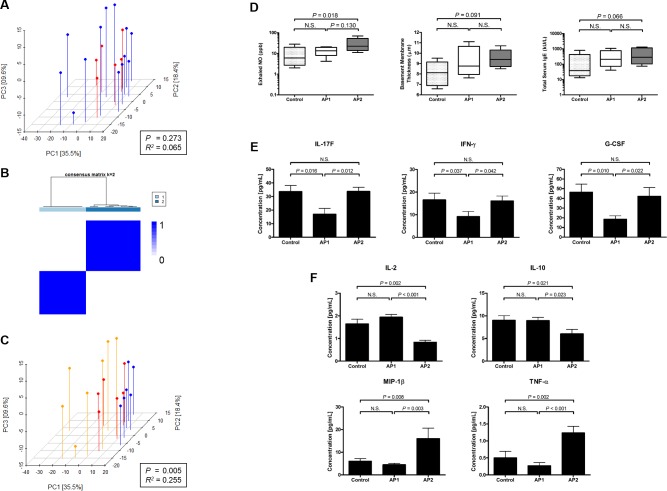
Cytokines and chemokines identify unique asthmatic phenotypes which are distinct from controls. (A) Principal coordinate analysis of 24 cytokines and chemokines measured in bronchoalveolar lavage (BAL) between control subjects (red) and asthmatics (blue). (B) Optimal clustering solution (k = 2) shown with heatmap of consensus indices (Legend: 1 = dark blue, 0 = white). (C) Asthmatics were reclassified based on their phenotype, as determined by unsupervised clustering: control (red), asthmatic phenotype (AP)1 (blue), and AP2 (orange). Significance of grouping was determined by PERMANOVA: *P* < 0.05 was considered significant and *R*^*2*^ is goodness of fit. (D) Clinical markers, exhaled NO (left), bronchial basement membrane thickness (middle), and total serum IgE (right), of asthma severity between controls, AP1, and AP2. Boxplot represent the median and quartiles. Exhaled NO and total serum IgE were log_10_ transformed for normalization. Significance determined by one-way ANOVA and Tukey’s post-hoc *t*-test, *P* < 0.05 was considered significant. BAL cytokines and chemokines were compared between controls and asthmatic phenotypes by pair-wise comparisons using *limma*. Cytokines and chemokines are shown that were found to be significantly (*P* < 0.05, *Q* < 0.1) enriched or depleted in (E) AP1 and (F) AP2. Each group is represented as mean and error bars are standard error of mean.

We first assessed markers of inflammation between asthmatics and controls. We analyzed bronchoalveolar lavage (BAL) cytokines and chemokines associated with T-cell responses and found no significant differences between asthmatics and controls using principal coordinate analysis (PCoA) and permutational MANOVA (PERMANOVA) (Fig 1A). We next queried whether sub-classifications of asthmatics were present utilizing unsupervised hierarchical clustering (S1 Fig). Two clusters of asthmatics were identified. We termed these clusters asthma phenotype (AP) 1 and AP2. AP1 and AP2 were found to have high within cluster consensus (AP1: 0.99 [95% CI 0.99–1.00], AP2: 0.97 [95% CI 0.96–0.98] Consensus Index) and low out of cluster consensus (0.04 [95% CI 0.02–0.05] Consensus Index) (Fig 1B). Using the new classification, there was significant separation between controls, AP1, and AP2 utilizing PCoA and PERMANOVA (*Pseudo-F* test, *P* = 0.005) (Fig 1C).

The immunologic phenotypes, AP1 and AP2, differed in pulmonary function. AP2 was significantly more obstructed with a lower FEV_1_ (% predicted). More individuals had mild or moderate obstruction compared to AP1 (Table 1). Both phenotypes had positive bronchial provocation tests with AP2 individuals requiring less mannitol to induce airway hyper-reactivity indicating more airway hyper-responsiveness in AP2 individuals. Though there were no significant increases in AP2 compared to both control and AP1, there was a significant linear trend to have higher fractional exhaled nitric oxide (geometric mean (GM) [95% CI]: 7[2–18] vs. 13[7–25] vs. 24[12–47] (ppb), control vs AP1 vs. AP2, *P* = 0.007), basement membrane thickness (mean [SD]: 8.1[1.0] vs. 9.1[1.2] vs. 9.5[0.7] (μM), control vs AP1 vs. AP2, *P* = 0.038), and total serum IgE (GM [95% CI]: 51[11–229] vs. 204[60–698] vs. 303[88–1043] (kU/L), control vs AP1 vs. AP2, *P* = 0.027), suggesting a more severe asthmatic phenotype (Fig 1D). With regards to age, sex, or body mass index (BMI), there were no significant differences between controls, AP1, or AP2 (Table 1).

**Table 1:** Clinical and pulmonary function primary baseline parameters.

		Control	AP1	AP2	*P*
**N**		6	6	7	
**Age**		23.3 (4.1)	22.2 (3.7)	20.1 (2.0)	0.298
**Male/Female**		4/2	2/4	4/3	0.489 *
**BMI**					0.251 *
	**<18.5**	0.00 (0)	0.00 (0)	0.00 (0)	
	**18.5–24.9**	66.7 (4)	33.3 (2)	71.4 (5)	
	**25.0–29.9**	33.3 (2)	16.7 (1)	14.3 (1)	
	**≥ 30.0**	0.00 (0)	50.0 (3)	14.3 (1)	
**FEV**_**1**_ **(L)**		3.9 (0.5)	3.5 (0.4)	3.1 (0.5)	0.063
**FEV**_**1**_ **(% predicted)**		94.6 (7.9)	89.8 (8.5)	76.4 (8.1)	0.006
**FVC (L)**		4.7 (0.6)	4.5 (0.7)	4.3 (0.9)	0.738
**FEV**_**1**_**/FVC**		0.82 (0.05)	0.79 (0.10)	0.73 (0.07)	0.129
**Obstruction FEV**_**1**_**/FVC < LLN**					0.042 *
	**Normal**		66.7 (4)	14.2 (1)	
	**Mild**		33.3 (2)	42.9 (3)	
	**Moderate**		0.00 (0)	42.9 (3)	
**PD15+**		0.00 (0)	100.0 (6)	100.0 (7)	<0.001 *
**PD15 (mg)**			255(112–580)	73 (40–134)	0.012
**ACQ Score**					0.521 *
	**<0.75**		50.0 (3)	33.3 (2)	
	**0.75–1.5**		16.7 (1)	16.7 (1)	
	**>1.5**		33.3 (2)	50.0 (3)	
**Bronchodilator Response**					0.758 *
	**ΔFEV**_**1**_ **(L) < 0.2 L**	83.3 (5)	66.7 (4)	66.7 (4)	
	**ΔFEV**_**1**_ **(L) > 0.2 L**	16.7 (1)	33.3 (2)	33.3 (2)	
**SPT+**		50.0 (3)	100.0 (6)	100.0 (7)	0.021 *

Continuous variables reported as mean (standard deviation) and categorical variable reported as percentage (N). Asthma phenotype (AP) 1 and 2, body mass index (BMI), forced expiratory volume in 1 second (FEV_1_), forced vital capacity (FVC), and lower limit of normal as determined by ATS/ERS guidelines 5] (LLN), positive mannitol bronchial provocation test (PD15 +), provocation dose mannitol bronchial provocation test (PD15), asthma control questionnaire (ACQ), positive skin prick test (SPT+).

* Significance determined by Chi-square analysis with boot-strap approximation of distribution

Significant differences in concentrations of BAL cytokines and chemokines were determined between controls, AP1, and AP2. AP1 had significantly decreased concentrations of G-CSF, IFN-γ, and IL-17F compared to AP2 and controls (Fig 1E, Table A in S1 File). Conversely, AP2 had significantly decreased IL-2 and -10 compared to both controls and AP1. AP2 also displayed increased concentrations of MIP-1β (CCL4) and TNF-α (Fig 1F, Table A in S1 File).

### Differentially expressed cytokine associate with airway obstruction

**Fig 2:**
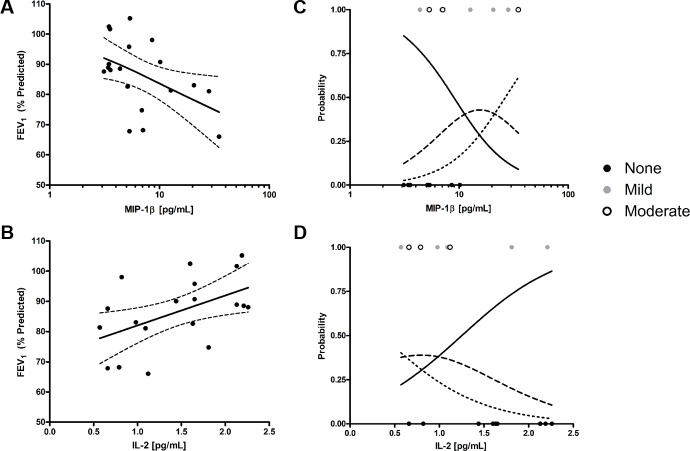
Asthmatic phenotypes have markers of increasing severity that is associated with MIP-1β and IL-2. Linear regression of (A) MIP-1β and (B) IL-2 associations with FEV_1_ (% predicted). Ordinal logistic regression of (C) MIP-1β and (D) IL-2 predicting type of obstruction: none or no obstruction (solid), mild obstruction (FEV_1_ (% predicted) > 70%) (dashed), moderate obstruction (FEV_1_ (% predicted) < 70%) (dotted). Solid and dashed lines for all graphs depict trend lines and 95% confidence intervals, respectively. All models were univariate. *P* < 0.05 was considered significant for the whole model and linear predictor.

We assessed whether the cytokines and chemokines that were differentially abundant between controls, AP1, and AP2 were also associated with the enhanced airway obstruction noted in AP2. To test for associations with airway obstruction, we constructed models where cytokine or chemokine concentrations were used as linear predictors of FEV_1_ (% predicted). IL-2 (*β* [95% CI] = 9.922[1.243–18.600], *R*^*2*^ = 0.255, *P* = 0.027) and MIP-1β (*β* [95% CI] = -17.220[-33.480–-0.921], *R*^*2*^ = 0.226, *P* = 0.039) significantly associated with FEV_1_ (% predicted) using univariate linear regression (Fig 2A and 2B). To confirm that these were not only correlated with low FEV_1_ (% predicted) in non-obstructed individuals, ordinal probabilistic models were constructed for no, mild, and moderate obstruction. Both inflammatory mediators, IL-2 (*β* [95% CI] = -1.841[-3.911–-0.160], *P* = 0.047) and MIP-1β (*β* [95% CI] = 3.861[0.897–7.471], *P* = 0.018) were significantly associated with the type of obstruction (Fig 2C and 2D) and suggest a role for IL-2 and MIP-1β in defining obstructive airway status.

### Altered cytokine concentrations and reduced obstruction is observed after inhaled corticosteroids

**Fig 3:**
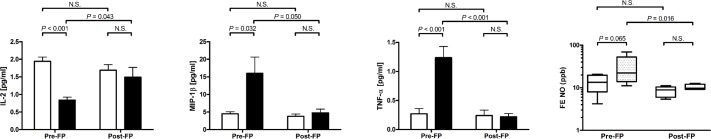
Inhaled fluticasone propionate increases IL-2 and decreases MIP-1β, TNF-α, and exhaled NO. Comparison of IL-2, MIP-1β, TNF-α, and exhaled NO between AP1 and AP2 pre- and post-six weeks of fluticasone propionate (FP) treatment. Comparison was performed using two-way ANOVA and Tukey’s post-hoc *t*-test, *P* < 0.05 was considered significant. Cytokines shown as mean and error bars represent standard error of mean. Boxplot represent the median and quartiles. Exhaled NO was log_10_ transformed for normalization.

After six weeks of therapy with fluticasone propionate (FP) individuals in both phenotypes had improved lung function with all but one individual in each group demonstrating airflow obstruction (Table B in S1 File). There was no significant difference between AP1 and AP2 with regards to pulmonary function or symptom control after FP treatment. This improvement in lung function corresponded to significantly (Tukey Post-hoc T-test, *P* < 0.05) increased concentrations of IL-2 and decreased concentrations of MIP-1β, TNF-α, and fractional exhaled nitric oxide in AP2 but not AP1 (Fig 3). There was no significant increase or reduction in IL-10, G-CSF, IFN-γ, or IL-17F (data not shown).

### Cytokine modules associate with asthmatic phenotypes and pulmonary function

**Fig 4:**
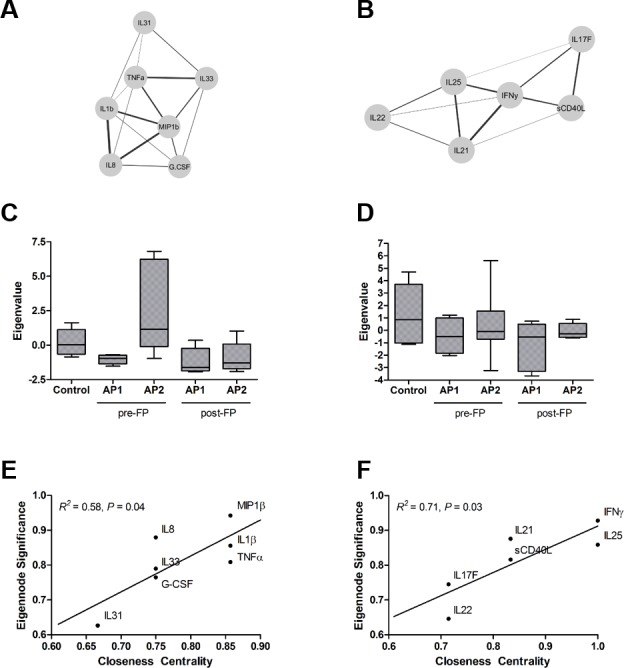
MIP-1β is hub in a cytokine module associated with AP2 pre-FP. Two modules, (A) Module 1 and (B) Module 2, were identified after clustering of cytokines with significant associations. Eigenvalue, linear transformation of cytokine abundances in each module, for (C) Module 1 and (D) Module 2. Hub cytokines were identified by eigennode significance and closeness centrality for (E) Module 1 and (F) Module 2.

To determine if the cytokines that were differentially abundant between controls and asthmatic phenotypes have effects that are independent or are correlated with other cytokines, we constructed a correlation network of cytokines across all subject samples (n = 30) using significant associations (Pearson’s correlations *P* < 0.05, *Q* < 0.05) (S2 Fig). We identified two modules of cytokines, Module 1 and Module 2 (Fig 4A and 4B). Module 1 was comprised of IL-1β, -8, -31, -33, TNF-α, G-CSF, and MIP-1β. Module 2 was comprised of IL-17F, -21, -22, -25, sCD40L, and IFN-γ. Module 1 abundance significantly varied with controls and asthmatic phenotypes pre- and post-FP (ANOVA *P* = 0.004), whereas Module 2’s variance was not significantly associated with controls and asthmatic phenotypes pre- and post-FP (ANOVA *P* = 0.349) (Fig 4C and 4D). In addition to Module 1 having increased abundance in AP2 pre-FP, Module 1 had a significant negative association with both FEV_1_ (% predicted) and FEV_1_/FVC (Table 2). Module 2 had a significant positive association with exhaled NO. Neither module had associations with basement membrane thickness. When assessing for hub cytokines within each module, we identified both MIP-1β and IFN-γ to have the highest correlation (eigennode significance) with the abundance of their modules and influence over their module (closeness centrality) (Fig 4E and 4F).

**Table 2:** Module associations with clinical and pulmonary parameters.

		Module 1* *P*	+/-	Module 2* *P*	+/-
**Unadjusted**	** **				
	**FEV**_**1**_ **(%Predicted)**	0.022	-	0.345	
	**FEV**_**1**_**/FVC**	0.012	-	0.327	
	**BMT (μm) †**	0.252		0.207	
	**Exhaled NO (ppb)**	0.545		0.009	+
**Adjusted for ICS ‡**	** **				
	**FEV**_**1**_ **(%Predicted)**	0.037	-	0.163	
	**FEV**_**1**_**/FVC**	0.022	-	0.11	
	**BMT (μm) †**	0.196		0.362	
	**Exhaled NO (ppb)**	0.972		0.009	+

Associations between modules and clinical or pulmonary parameters using linear regression for all individuals (n = 30).

* Eigen Value Module 1 determined by abundances of IL-1β, IL-8, IL-31, G-CSF, TNF-α, and MIP-1β. Module 2 determined by abundances of IL-17F, IL-21, IL-22, IL-25, sCD40L, and IFN-γ.

† Basement membrane thickness (BMT).

‡ Linear regression adjusted for inhaled corticosteroids (FP) usage and the interaction term between module and ICS. Sign designates the whether it is a positive (+) or negative (-) association.

### The airway microbiome varies between asthmatic phenotypes, non-asthmatics, and after inhaled corticosteroid treatment

We hypothesized that the difference in cytokine profile in BAL was related to microbial composition that could be detected in the BAL. To test this we utilized shotgun sequencing of BAL to determine the composition of the microbiome. We detected 656 taxa at the species level with mean read count of one across all samples. We assessed for differences in α-diversity across samples (S3A Fig). We found no difference between phenotypes and controls. There was a significant increase in diversity after corticosteroid treatment in both phenotypes. To assess between group diversity (β-diversity), taxa counts were normalized using median sum scaling (see Methods) and fit onto detrended correspondence analysis to determine if there was separation of samples along species gradients (S3B Fig). There was significant separation of the phenotypes along the first correspondence axis (DC1) (Table C in S1 File).

**Fig 5:**
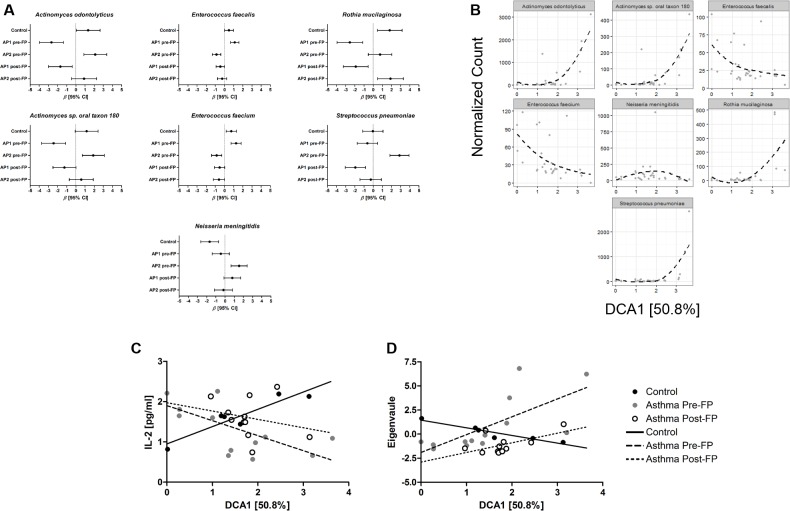
The changes in airway microbiome have unique associations with inflammation in asthmatics. (A) *β* coefficients and 95% CI from negative binomial generalized linear model fits for each differentially abundant species in controls and asthmatic phenotypes pre- and post-FP. (B) LOEWSS regressions of normalized counts of differentially abundant species and DCA1. Univariate linear regressions of (C) IL-2 and (D) Module 1 using DCA1 as a linear predictor. Line type and point color represents controls, asthmatics pre-, or post-FP.

To test which taxa were differentially abundant between controls, AP1, and AP2, normalized taxa counts were fit to generalized linear models using the negative binomial distribution (S4 Fig) and sample classification (control, AP1 pre-FP, AP2 pre-FP, AP1 post-FP, and AP2 post-FP) as linear predictors (Table D in S1 File). A trend toward higher error was noted with taxa with low fitted counts, likely due to insufficient modeling of excess zeroes (S4C Fig). We therefore choose to focus our analysis on the top 25 most abundant species, which all had a mean abundance greater that 30 normalized counts. We detected seven species that were differentially abundant (FDR adjusted *P* < 0.05) between controls, AP1 pre-FP, and AP2 pre-FP (Fig 5A).

An increased abundance of *Streptococcus pneumoniae* was found in AP2 pre-FP compared to AP1 pre-FP (Log_2_ Fold Change (L2FC) [95% CI]: 3.504[1.815–5.192], adjusted *P* = 0.002, AP2/AP1) and controls (L2FC [95% CI]: 2.909[1.219–4.598], adjusted *P* = 0.002, AP2/control). There was also a trend toward increased abundance in *Neisseria meningitidis* in AP2 pre-FP compared to AP1 pre-FP (L2FC [95% CI]: 1.998[0.586–3.409], adjusted *P* = 0.055, AP2/AP1) and controls (L2FC [95% CI]: 3.216[1.774–4.659], adjusted *P* = 0.002, AP2/control). The increase in these pathogenic species was not observed in AP1 pre-FP (*S*. *pneumoniae* adjusted *P* = 0.816 and *N*. *meningitidis* adjusted *P =* 0.654). There was decreased abundance of *Rothia mucilaginosa*, *Actinomyces odontolyticus*, and *Actinomyces sp*. *oral taxon 180* in AP1 compared to controls and AP2. Whereas, AP2 had decreased abundance of *Enterococcus faecalis* and *faecium* compared to AP1 and controls (Tables E-G in S1 File).

After six weeks of FP treatment there were significant reductions in the abundance of *S*. *pneumoniae*, *N*. *meningitis*, *E*. *faecium*, and *E*. *faecalis* (Fig 4). AP1 showed a significant reduction in *E*. *faecalis* (L2FC [95% CI]: -1.576[-0.854–2.298], *P* < 0.001, AP1 pre-FP/post-FP) and *E*. *faecalis* (L2FC [95% CI]: -1.802[-2.987–4.277], *P* < 0.001, AP1 pre-FP/post-FP). AP2 had a significant decrease in abundance of *S*. *pneumoniae* (L2FC [95% CI]: -3.139[-1.390–4.889], *P* < 0.001, AP2 pre-FP/post-FP) and *N*. *meningitis* (L2FC [95% CI]: -1.708[-0.241–3.174], *P =* 0.022, AP1 pre-FP/post-FP). There was no significant change in *R*. *mucilaginosa*, *A*. *odontolyticus*, and *A*. *sp*. *oral taxon 180* in either phenotype after fluticasone propionate treatment (Tables H-J in S1 File).

### The airway microbiome is associated with differential cytokine production observed in asthmatics

To further assess if the differences in the microbiome were related to differences in cytokines we performed multiple regressions with DC1 as a linear predictor for cytokines in controls, asthmatics pre-FP, and asthmatics post-FP. DC1 represents a gradient for the differentially abundant species identified using generalized linear models (Fig 5B). Notably, *E*. *faecalis* and *E*. *faecium* decrease along the gradient, whereas *A*. *odontolyticus*, *A*. *sp*. *oral taxon 180*, *R*. *mucilaginosa*, and *S*. *pneumoniae* increased along the gradient. The relationship between cytokines and DC1 was significantly different for IL-2, IFN-γ, TNF-α, and Module 1 (ANCOVA *P* = 0.006, 0.030, 0.002, 0.007, respectively) when comparing controls, asthmatics pre- and post-FP (Table 3). There were also trends in G-CSF, MIP-1β, and Module 2 (ANCOVA *P =* 0.096, 0.058, respectively). IL-2 had a significant negative association with DC1 in asthmatics pre-FP not seen in controls or asthmatics post-FP. Conversely, Module 1 had significant positive associations with DC1 in asthmatics pre-FP not seen in controls or asthmatics post-FP (Fig 5C and 5D). Differences in microbiome composition, pulmonary function, and cytokine concentrations were not attributable to differences in FP dosing (S5 Fig).

**Table 3:** Associations between differentially expressed cytokines, cytokine module, and DC1.

		*β*	*95% CI*	*R*^*2*^	*F*	*P*	ANCOVA *P*
**IL-2**							0.006
	**Control**	0.43	0.190–0.668	0.862	24.89	0.008	
	**Asthma Pre-FP**	-0.373	-0.661–0.084	0.424	8.093	0.016	
	**Asthma Post-FP**	-0.208	-0.833–0.418	0.059	0.563	0.472	
**IL-10**							0.508
	**Control**	-0.845	-3.887–2.197	0.129	0.594	0.484	
	**Asthma Pre-FP**	-0.615	-2.158–0.928	0.065	0.77	0.399	
	**Asthma Post-FP**	-2.264	-4.113–0.415	0.46	7.676	0.022	
**IL-17F**							0.341
	**Control**	2.351	-11.17–15.87	0.055	0.233	0.655	
	**Asthma Pre-FP**	8.42	3.323–13.520	0.546	13.22	0.004	
	**Asthma Post-FP**	-1.021	-19.65–17.61	0.002	0.015	0.904	
**G-CSF**							0.096
	**Control**	-8.322	-31.53–14.89	0.199	0.991	0.376	
	**Asthma Pre-FP**	11.31	0.525–22.090	0.326	5.328	0.041	
	**Asthma Post-FP**	4.603	-14.29–23.5	0.033	0.304	0.595	
**IFN-γ**							0.03
	**Control**	-3.614	-11.56–4.327	0.285	1.596	0.275	
	**Asthma Pre-FP**	4.008	1.103–6.914	0.456	9.219	0.011	
	**Asthma Post-FP**	2.421	-4.196–9.037	0.071	0.685	0.429	
**MIP-1β**							0.058
	**Control**	-1.668	-4.303–0.967	0.436	3.087	0.154	
	**Asthma Pre-FP**	5.774	0.608–10.940	0.355	6.053	0.032	
	**Asthma Post-FP**	1.295	-1.119–3.708	0.141	1.472	0.256	
**TNF-α**							0.002
	**Control**	-0.143	-0.708–0.422	0.11	0.496	0.52	
	**Asthma Pre-FP**	0.478	0.257–0.699	0.673	22.66	0.001	
	**Asthma Post-FP**	-0.051	-0.288–0.187	0.025	0.234	0.64	
Eigen Value Module 1*							0.007
	**Control**	-0.797	-1.151–0.443	0.907	39.13	0.003	
	**Asthma Pre-FP**	1.85	0.601–1.028	0.492	10.63	0.008	
	**Asthma Post-FP**	1.001	-0.035–2.036	0.347	4.779	0.057	
Eigen Value Module 2*							0.078
	**Control**	1.256	-1.017–3.528	0.37	2.352	0.199	
	**Asthma Pre-FP**	-0.892	-2.066–0.281	0.203	2.801	0.122	
	**Asthma Post-FP**	-0.32	-2.225–1.585	0.016	0.145	0.713	

*Eigen Value Module 1 determined by abundances of IL-1β, IL-8, IL-31, G-CSF, TNF-α, and MIP-1β. Eigen Value Module 2 determined by abundances of IL-17F, IL-21, IL-22, IL-25, sCD40L, and IFN-γ.

## Discussion

Within a homogenous group of atopic asthmatics (i.e. positive skin prick tests, elevated total serum IgE, and elevated exhaled NO), we identified two distinct asthmatic phenotypes (AP) termed AP1 and AP2. These are associated with differences in the local inflammatory milieu, lower airway microbiota composition, and pulmonary function. We identified MIP-1β and IL-2 as cytokines associated with the obstructive status in asthmatics. The abundance of these cytokines was associated with composition of the microbiome. IL-2 dominant profiles were associated with increased *Enterococcus* species, whereas MIP-1β dominant profiles were associated with *Streptococcus pneumoniae* and Oral taxa. We observed a reduction in MIP-1β and an increase in IL-2 after inhaled corticosteroid therapy. Additionally, the associations between the microbiome composition and cytokine profiles were diminished after inhaled corticosteroid therapy.

Interestingly, with regards to baseline pulmonary function, AP1 and AP2 resemble cluster 1 and 2 from Moore WC et al. (2010). In this large analysis, asthma phenotypes were identified using unsupervised clustering of asthmatics based on clinical parameters [3]. All individuals in our study had onset of asthma in childhood, positive skin prick tests, and elevated IgE, which is consistent with atopic asthma and the individuals identified in cluster 1 and 2. Similar to cluster 1, AP1 had preserved baseline pulmonary function parameters (FEV_1_ (% predicted): 102 vs. 90, and FEV_1_/FVC: 0.78 vs. 0.79, cluster 1 vs. AP1), whereas AP2 moderately reduced pulmonary function baseline more comparable to cluster 2 (FEV_1_ (% predicted): 82 vs. 76, and FEV_1_/FVC: 0.74 vs. 0.73, cluster 2 vs. AP2). Importantly, this suggests a link between pulmonary function and the immunology of the airway as we had defined these clusters based on immunologic profiles. In asthmatics prior to treatment the immunologic profiles were highly associated with the composition of the microbiota in airway. These data indicate that in atopic asthmatics differences in pulmonary function may be mediated by unique immune responses to the airway microbiome.

AP1 exhibited significantly decreased concentrations of cytokines (IFN-γ, IL-17F, and G-CSF) associated with microbial pathogens [12, 18]. In contrast, AP2 had decreased concentrations of cytokines associated with reduced airway inflammation (IL-2 and IL-10) [9, 32, 33]. Additionally, AP2 exhibited higher concentrations of TNF-α and MIP-1β. Notably, the concentrations of IL-2 and IL-10 were independent of other cytokines. In contrast, TNF-α and MIP-1β were highly correlated with IL-1β, -8, -31, and -33, which we identified as Module 1. MIP-1β was identified as a hub in Module 1. The pro-inflammatory Module 1 was associated with a greater likelihood of airway obstruction, negatively correlating FEV_1_ (% predicted) and FEV_1_/FVC. Importantly, IL-8, -31, and -33 have been shown to negatively correlate in independent associations with FEV_1_ (% predicted) [34–36]. The abundance of Module 1 was reduced by treatment with the inhaled corticosteroid, fluticasone propionate. Future investigations may identify specific cell populations secreting cytokines related to changes in the microbiome.

The composition of the airway microbiome also significantly differed between asthmatic phenotypes. AP2 was enriched with species from the upper airways, *Actinomyces ondontolyticus*, *Actinomyces oral taxon 180*, *Neisseria menigiditis*, and *Streptococcus pneumoniae*, whereas AP1 was enriched with *Enterococcus* species. These species varied along the constructed ecological gradient DCA1. Similar to previous reports, these data suggest that the lung microbiome can vary with regards to abundance of upper airway taxon [17, 18]. Interestingly, asthmatic individuals may have impaired mucocilliary clearance upon allergen exposure and therefore might have different rates of elimination of upper-airway species [37]. Unlike what has been previously reported in non-asthmatic individuals, in asthmatics prior to FP treatment the increasing abundance of these upper airway species was not associated with Th17 responses but was highly associated with decreasing IL-2 and increasing Module 1.

Interestingly, the abundance of *Streptococcus pneumoniae* was reduced after FP treatment which correlated with the reduction in concentration of Module 1 (IL-1β, -8, -31, -33, TNF-α and MIP-1β). Previously, the abundance of *Streptococcus* in sputum has been shown to positively correlate with IL-8 and neutrophils [35]. *Actinomyces* and *Neisseria* species have been shown to be enriched in asthmatics but also increased in abundance with lower BAL eosinophils [20]. In our study, DCA1 represented an increasing gradient of *Actinomyces ondontolyticus*, *Actinomyces oral taxon 180*, *Neisseria menigiditis*, and *Streptococcus pneumoniae*. DCA1 had unique associations with IL-2 and Module 1 in asthmatics prior to FP treatment. In murine infection models, *Streptococcus pneumoniae* induces increased production of many of the cytokines in Module 1 [38].

Our study, though limited in sample size and racial diversity, highlights the heterogeneous nature of asthma. Even within asthmatics that fit the picture of atopic asthma, there appears to significant variation within local inflammation and the lower airway microbiome, thus allowing us to discern two distinct phenotypes. Our observation may have been strengthened by the strong exclusion criteria: no history of smoking in the past year, less than a five pack year history of smoking, and no symptoms of respiratory infection in the proceeding 6 weeks. Although the inclusion and exclusion criteria control for several potential confounders, the small sample size of this study limits the data to be less generalizable to all asthmatics and effects covariates associated with disease severity might be missed, such as BMI which made up 50% of AP1 subjects. In addition to larger sample sizes, future investigations of smokers and those after an infection or asthma exacerbation would be of interest to test for subtypes similar to AP1 and AP2.

In addition to small sample size, our analysis did not include assessment of BAL cellular profiles (e.g. % of eosinophils in BAL) nor did it include a placebo arm for treatment comparison. It should be noted that BAL cellular profile are associated with differences in the airway microbiome [39, 40]. Notably, in these studies the taxa *Streptoccocus*, *Neisseria*, *Actinomyces*, and *Rothia* were identified as differentially abundant between asthmatics with different granulocytic profiles. The lack of placebo arm reduces conclusions that can be drawn after steroid treatment, as it is not known whether the changes in the microbiome or inflammation are due to random temporal exposures to bacteria. Due to clinical limitations of obtaining serial samples from the same individuals, this area of literature is lacking. The association between the exposure to different microbiota and the detected inflammation was not significant after treatment (Table 3). These data suggest that even if the bacterial variation is due random temporal variation, ICS therapy may reduce the inflammation associated with exposure to specific bacterial species, such as those from the oral cavity.

Currently, more personalized approaches are being developed for treatment of the most severe forms of asthma. Our study indicates that even in mild to moderate asthmatics with intermittent or persistent symptom there are distinct sub-phenotypes (AP1 and AP2) that differ in their immune response and microbiome. These sub-phenotypes are associated with important aspects of airway physiology. Additionally, monitoring for microbiological changes, such as an abundance of *Streptococcus pneumoniae* and oral-pharyngeal taxa, may allow for personalized approaches for patient monitoring.

## Supporting information

S1 Fig Hierarchical clustering of asthmatic subjects pre-fluticasone propionate identifies two clusters. Bronchoalveolar lavage (BAL) cytokines and chemokines from asthmatic subjects were standardized using *z-*score and sample distances were determined using Euclidean distances. Sample wise distance matrix was used for metric multidimensional scaling and (A) a Scree plot of the components was used to determine how many components to use in the clustering algorithm. The first six components were selected (containing > 90% of all sample variance) and underwent hierarchical clustering using Pearson’s correlation’s as distances and Unweighted Pair Group Method with Arithmetic Mean (UPGMA) as the clustering algorithm. (B) Average silhouette width for each cluster solution shown with cross hairs showing optimal cluster solution. (C) Consensus clustering was used to determine stability of various clustering solution k = 2–8 (colors) and cumulative distribution functions of consensus indices for each solution are shown.
(TIF)

S2 Fig Correlation network of cytokines. (A) Heatmap showing significant (P < 0.05 and Q < 0.05) absolute Pearson’s correlation between cytokines. Cytokines were clustered using the complete linkage algorithm. (B) Correlation network of cytokines with significant associations with each other. Modules are highlighted by color, Module 1 (blue) and Module 2 (red). Size of edge is proportional to strength of association. Straight lines signify positive correlations and sine wave lines signify negative correlations.
(TIF)

S3 Fig α- and β-Diversity of broncho-alveolar lavage microbiome. (A) Rarefaction curves and (B) Detrendend correspondence analysis of 656 microbial species showing the first two scaled ordination axes, DCA1 and DCA2, which account for 50.8% and 25.9% of variance between all samples. Colors represent groups: control (light blue), AP1 pre-FP (black), AP2 pre-FP (green), AP1 post-FP (red), and AP2 post-FP (blue). Ellipses are standard deviation from centroids of each group. Significant factors and vectors plotted as text in red and determined centroids. Significance determined by *R*^*2*^ and degrees of freedom, *P <* 0.05 was considered significant.
(TIF)

S4 Fig Negative Binomial for modeling taxa counts. (A) Dispersion parameter for individual taxa (blue) is determined by initial estimation (black) and Bayesian shrinkage using the prior knowledge of local average regression of dispersion with mean of normalized count as the predictor (trend line, red). (B) Fitted counts plotted against observed counts with a pseudo-counts of one added to each to visualize zeros. (C) Residuals plot showing error vs. fitted counts with counts greater than 30 displaying a more normally distributed error. (D) Q-Q plot of observed quantiles versus the theoretical quantiles of random data generated using the negative binomial distribution and parameters for each taxa, *NB*(*μ =* mean, *α =* dispersion).
(TIF)

S5 Fig No difference in pulmonary function, cytokines, or microbiome between low and high dose fluticasone propionate. Boxplots of FEV_1_ (% predicted), FEV_1_/FVC, Module 1, IL-2, and DCA1 between individuals randomized to low (100 μg/BID) or high dose (500 μg/BID). *P* < 0.05 was considered significant, Welch’s T-test.
(TIF)

S1 File **Supplemental tables A-J.** Supplemental tables A-J.
(DOCX)
