# Supplementary figures and images for: Atopic asthmatic immune phenotypes associated with airway microbiota and airway obstruction
Source: PLoS One. 2017 Oct 20;12(10):e0184566. doi: 10.1371/journal.pone.0184566 (PMC5650135; doi:10.1371/journal.pone.0184566)

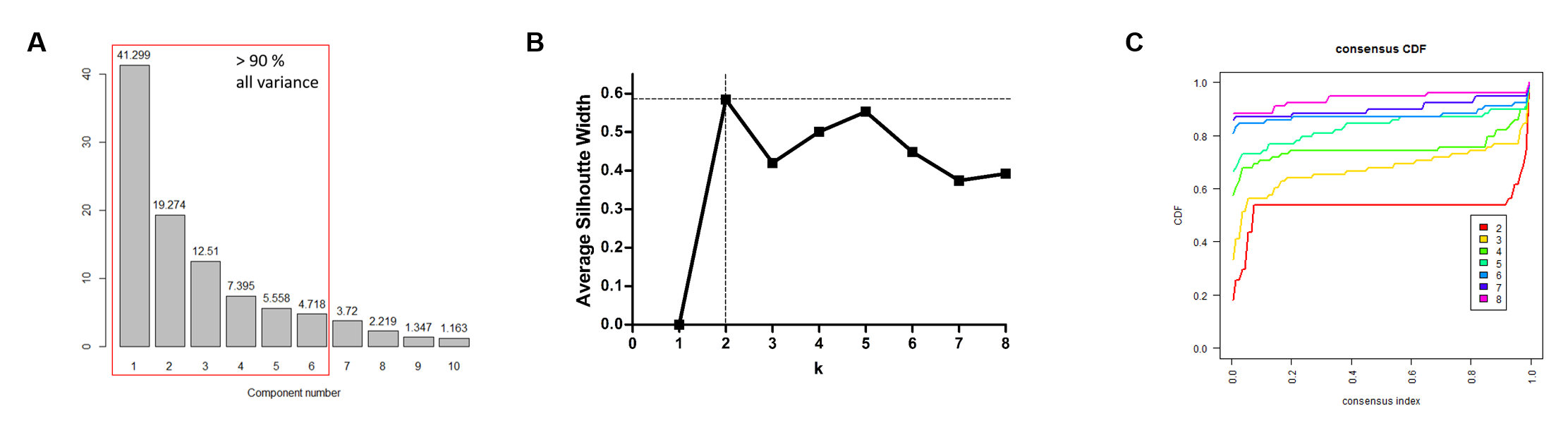

Supplement: S1 Fig — Bronchoalveolar lavage (BAL) cytokines and chemokines from asthmatic subjects were standardized using z-score and sample distances were determined using Euclidean distances. Sample wise distance matrix was used for metric multidimensional scaling and (A) a Scree plot of the components was used to determine how many components to use in the clustering algorithm. The first six components were selected (containing > 90% of all sample variance) and underwent hierarchical clustering using Pearson’s correlation’s as distances and Unweighted Pair Group Method with Arithmetic Mean (UPGMA) as the clustering algorithm. (B) Average silhouette width for each cluster solution shown with cross hairs showing optimal cluster solution. (C) Consensus clustering was used to determine stability of various clustering solution k = 2–8 (colors) and cumulative distribution functions of consensus indices for each solution are shown. (TIF) [file pone.0184566.s001.tif]

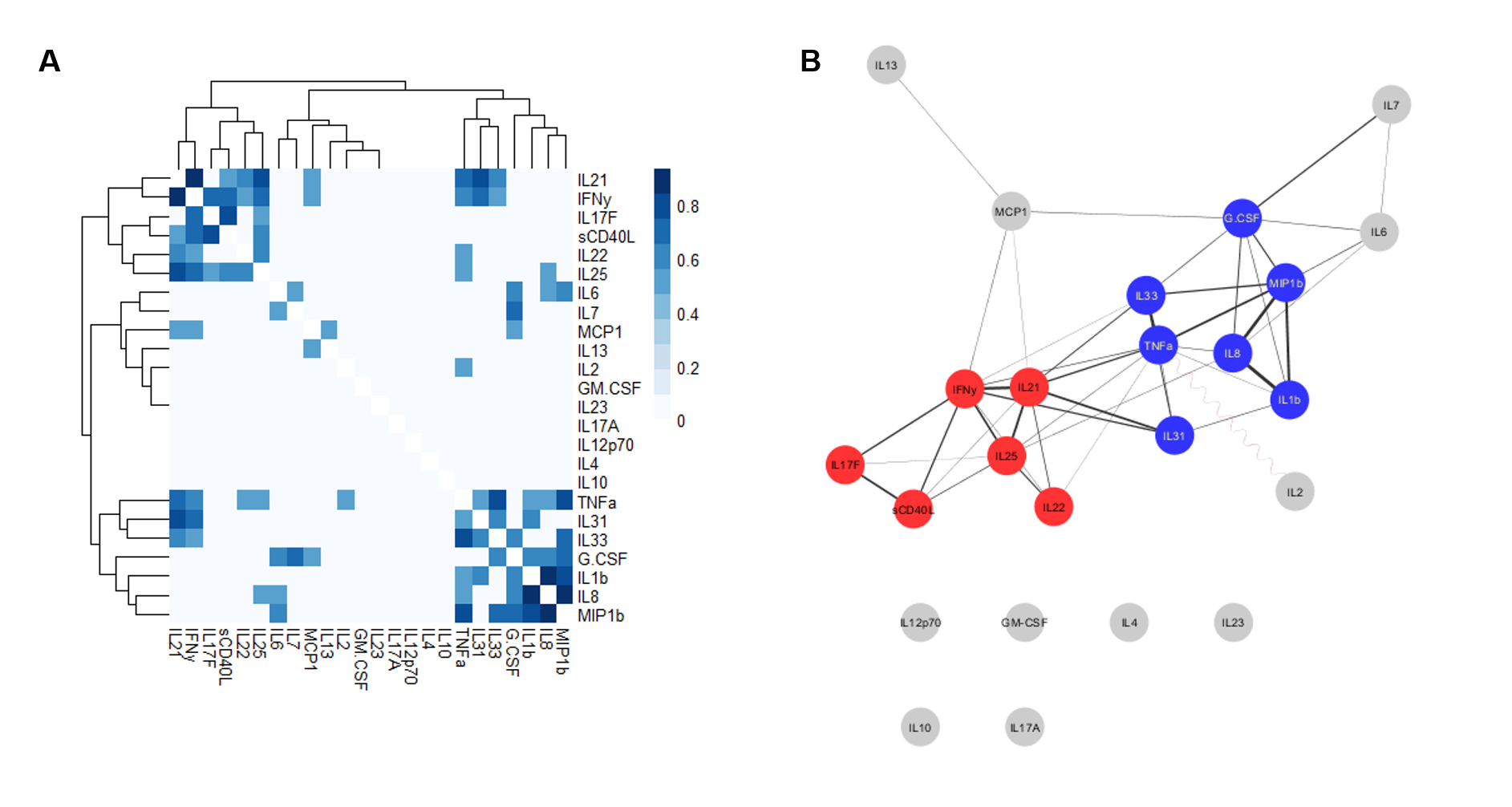

Supplement: S2 Fig — (A) Heatmap showing significant (P < 0.05 and Q < 0.05) absolute Pearson’s correlation between cytokines. Cytokines were clustered using the complete linkage algorithm. (B) Correlation network of cytokines with significant associations with each other. Modules are highlighted by color, Module 1 (blue) and Module 2 (red). Size of edge is proportional to strength of association. Straight lines signify positive correlations and sine wave lines signify negative correlations. (TIF) [file pone.0184566.s002.tif]

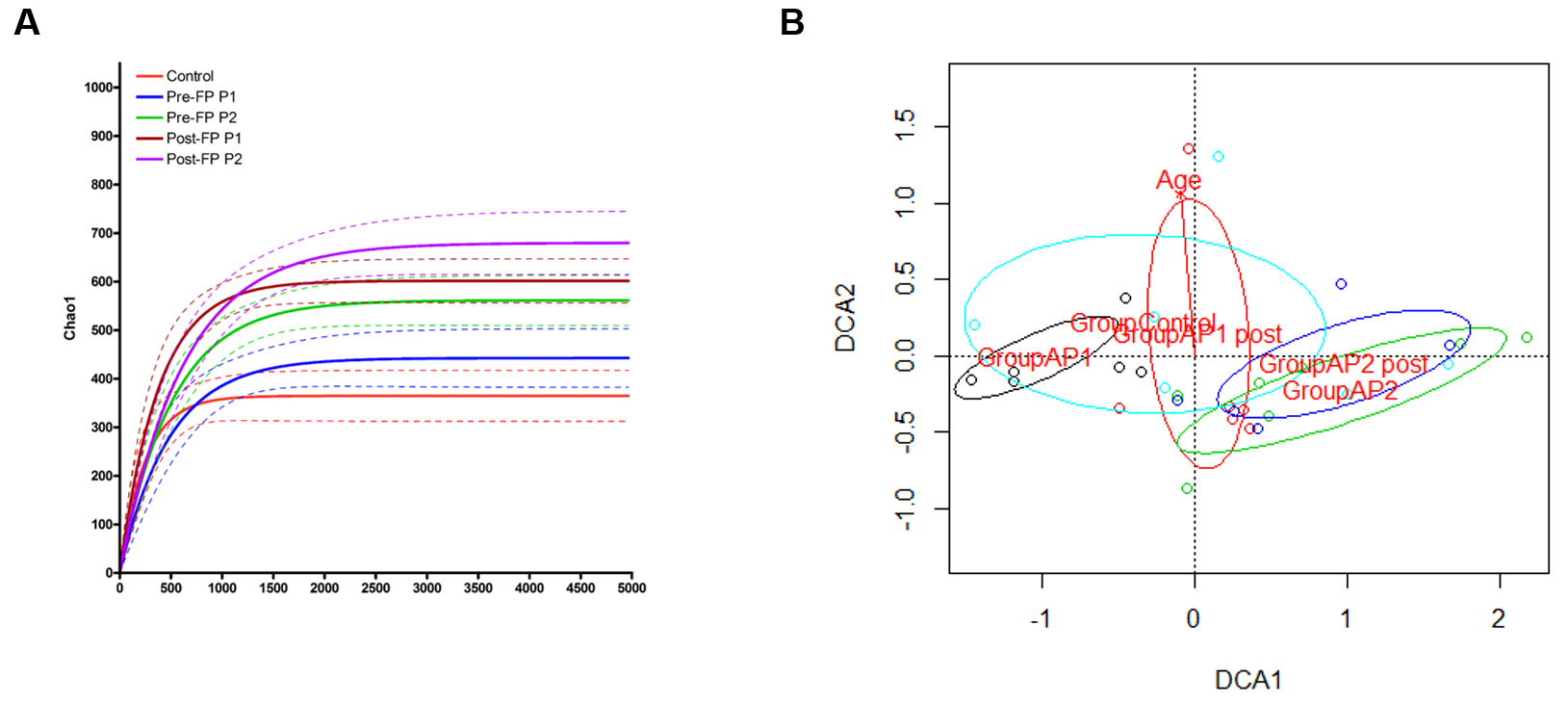

Supplement: S3 Fig — (A) Rarefaction curves and (B) Detrendend correspondence analysis of 656 microbial species showing the first two scaled ordination axes, DCA1 and DCA2, which account for 50.8% and 25.9% of variance between all samples. Colors represent groups: control (light blue), AP1 pre-FP (black), AP2 pre-FP (green), AP1 post-FP (red), and AP2 post-FP (blue). Ellipses are standard deviation from centroids of each group. Significant factors and vectors plotted as text in red and determined centroids. Significance determined by R2 and degrees of freedom, P < 0.05 was considered significant. (TIF) [file pone.0184566.s003.tif]

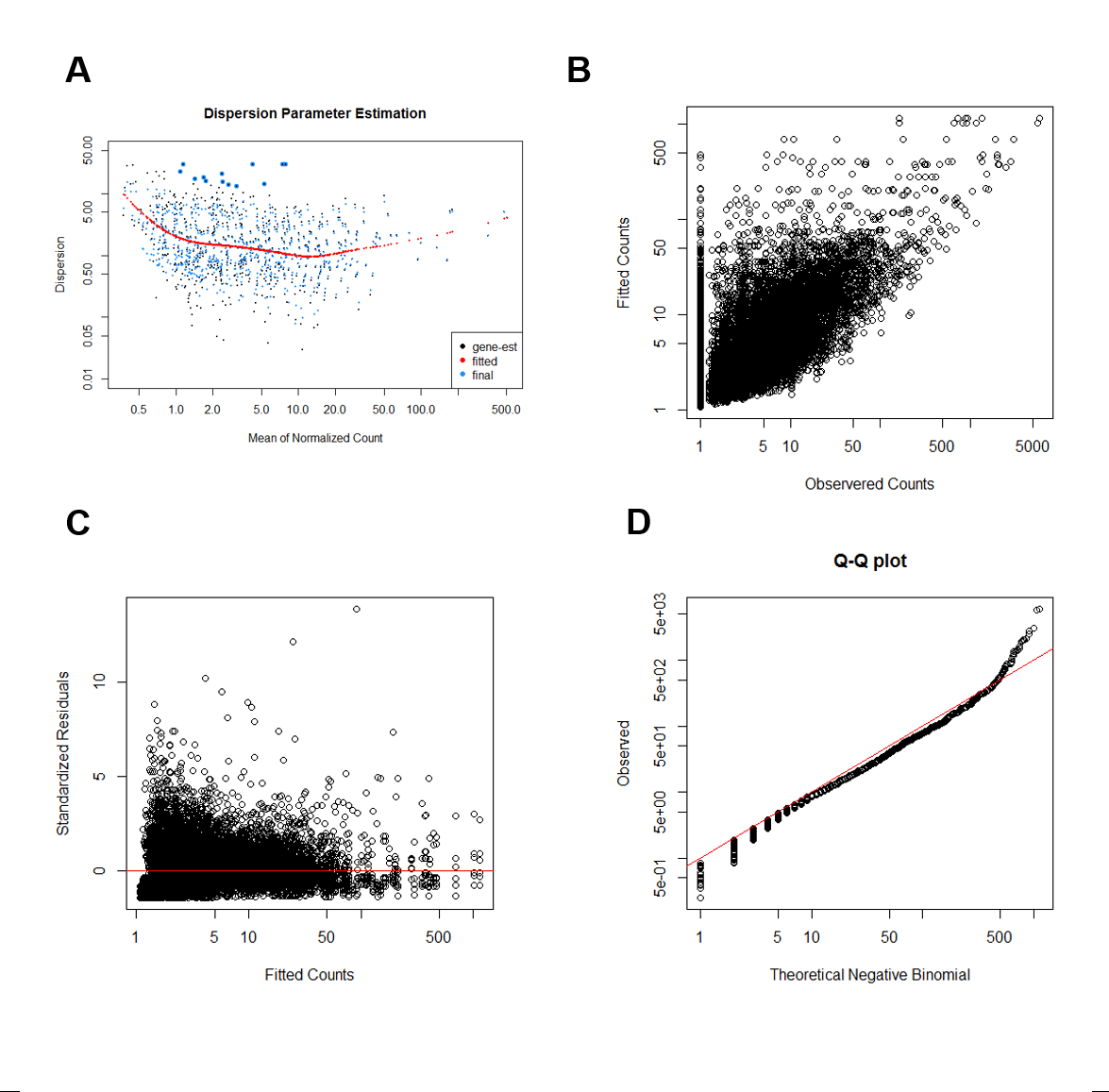

Supplement: S4 Fig — (A) Dispersion parameter for individual taxa (blue) is determined by initial estimation (black) and Bayesian shrinkage using the prior knowledge of local average regression of dispersion with mean of normalized count as the predictor (trend line, red). (B) Fitted counts plotted against observed counts with a pseudo-counts of one added to each to visualize zeros. (C) Residuals plot showing error vs. fitted counts with counts greater than 30 displaying a more normally distributed error. (D) Q-Q plot of observed quantiles versus the theoretical quantiles of random data generated using the negative binomial distribution and parameters for each taxa, NB(μ = mean, α = dispersion). (TIF) [file pone.0184566.s004.tif]

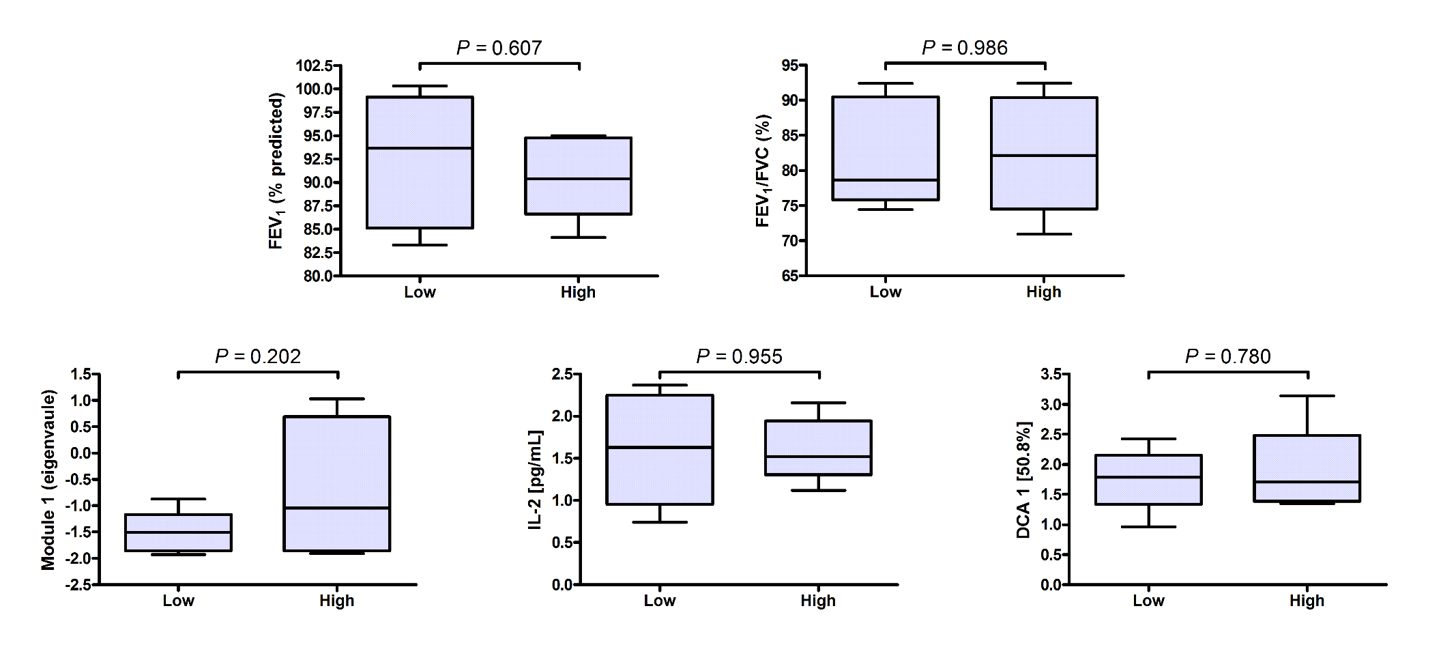

Supplement: S5 Fig — Boxplots of FEV1 (% predicted), FEV1/FVC, Module 1, IL-2, and DCA1 between individuals randomized to low (100 μg/BID) or high dose (500 μg/BID). P < 0.05 was considered significant, Welch’s T-test. (TIF) [file pone.0184566.s005.tif]
