# Supplementary material for: Atopic asthmatic immune phenotypes associated with airway microbiota and airway obstruction
Source: PLoS One. 2017 Oct 20;12(10):e0184566. doi: 10.1371/journal.pone.0184566 (PMC5650135; doi:10.1371/journal.pone.0184566)
Supplement: S1 File — Supplemental tables A-J. Supplemental tables A-J. (DOCX) [file pone.0184566.s006.docx]

**Supplemental Text**

**Table A. BAL Cytokine and Chemokines**

**C (pg/mL) AP1 (pg/mL) AP2 (pg/mL) C-AP1 *P* C-AP2 *P* AP1-AP2 *P***

**IL21** 51.39 (39.89) 24.21 (17.1) 53.82 (27.29) 0.115 (0.173) 0.881 (0.966) 0.077 (0.027)

**G-CSF** 46.43 (18.46) 18.54 (7.73) 42.25 (22.06) 0.010 (0.098) 0.671 (0.894) 0.022 (0.010)

**GMCSF** 45.26 (2.05) 43.08 (1.32) 41.02 (3.25) 0.171 (0.219) 0.009 (0.052) 0.180 (0.046)

**IL17F** 33.71 (9.9) 17.03 (9.49) 33.78 (7.58) 0.016 (0.098) 0.991 (0.991) 0.012 (0.009)

**MCP1** 20.21 (3.93) 17.49 (1.7) 19.9 (1.79) 0.122 (0.173) 0.851 (0.966) 0.154 (0.042)

**IFN-γ** 16.58 (6.69) 9.18 (5.06) 16.12 (5.24) 0.037 (0.098) 0.887 (0.966) 0.042 (0.016)

**IL23** 15.77 (7.89) 18.6 (10.72) 3.42 (6.02) 0.632 (0.429) 0.037 (0.111) 0.012 (0.009)

**IL8** 14.1 (8.07) 4.63 (2.46) 32.15 (35.01) 0.394 (0.360) 0.098 (0.262) 0.015 (0.009)

**sCD40L** 10.31 (2.02) 7.51 (2.82) 9.44 (1.67) 0.046 (0.098) 0.506 (0.867) 0.146 (0.042)

**IL17A** 9.25 (1.48) 9.84 (1.92) 9.13 (2.76) 0.611 (0.429) 0.916 (0.966) 0.527 (0.115)

**IL10** 9.01 (2.32) 8.96 (1.61) 6.05 (2.37) 0.967 (0.515) 0.021 (0.100) 0.023 (0.010)

**IL31** 8.5 (4.85) 4.66 (3.35) 10.06 (4.63) 0.302 (0.298) 0.662 (0.894) 0.137 (0.042)

**MIP1β** 6.04 (2.5) 4.5 (1.26) 16.0 (11.34) 0.671 (0.429) 0.008 (0.052) 0.003 (0.004)

**IL33** 4.97 (3.21) 4.45 (1.84) 9.53 (4.89) 0.801 (0.458) 0.028 (0.111) 0.015 (0.009)

**IL7** 3.49 (0.86) 1.97 (0.74) 2.62 (1.37) 0.037 (0.098) 0.202 (0.441) 0.337 (0.081)

**IL22** 2.69 (1.9) 3.38 (1.4) 3.24 (2.07) 0.482 (0.411) 0.559 (0.894) 0.884 (0.170)

**IL1β** 1.91 (1.92) 0.66 (0.16) 6.74 (6.58) 0.581 (0.429) 0.034 (0.111) 0.009 (0.009)

**IL2** 1.64 (0.46) 1.94 (0.27) 0.84 (0.21) 0.238 (0.277) 0.002 (0.029) 0.000 (0.000)

**IL4** 1.44 (0.23) 1.51 (0.07) 1.63 (0.25) 0.642 (0.429) 0.173 (0.415) 0.370 (0.085)

**IL6** 0.7 (0.33) 0.39 (0.27) 0.85 (0.37) 0.111 (0.173) 0.422 (0.844) 0.018 (0.009)

**IL25** 0.54 (0.45) 0.09 (0.19) 0.43 (0.46) 0.045 (0.098) 0.605 (0.894) 0.109 (0.036)

**TNFα** 0.5 (0.43) 0.27 (0.2) 1.24 (0.48) 0.303 (0.298) 0.002 (0.029) 0.000 (0.000)

**IL13** 0.25 (0.19) 0.22 (0.14) 0.24 (0.18) 0.824 (0.458) 0.925 (0.966) 0.891 (0.170)

**IL12p70** 0.1 (0.2) 0.16 (0.23) 0.22 (0.37) 0.721 (0.439) 0.484 (0.867) 0.741 (0.155)

Cytokine and Chemokine concentrations for Controls (C), asthma phenotype (AP) 1, and AP2 reported as mean (standard deviation) and significance of pairwise wise comparisons reported as *P* (*Q* value).

**Table B. Clinical and Pulmonary Function Primary Endpoint Parameter**

**AP1 AP2 *P***

**N** 6 6

**Male/Female** 2/4 4/2 0.248

**Fluticasone Propionate Dose** 0.558 *

**100 μg/BID** 50.0 (3) 33.3 (2)

**500 μg/BID** 50.0 (3) 66.6 (4)

**FEV_1_ (L)** 3.7 (0.4) 3.7 (0.5) 0.966

**FEV_1_ (L) MID** 0.558 *

**ΔFEV_1_ (L) <0.1L** 50.0 (3) 33.3 (2)

**ΔFEV_1_ (L) >0.1L** 50.0 (3) 66.7 (4)

**FEV_1_ (% predicted)** 93.4 (5.6) 87.7 (4.2) 0.103

**FVC (L)** 4.5 (0.7) 4.6 (0.7) 0.889

**FVC (L) MID** 0.221 *

**ΔFVC (L) <0.1L** 16.7 (5) 50.0 (3)

**ΔFVC (L) >0.1L** 83.3 (1) 50.0 (3)

**FEV_1_/FVC** 0.82 (0.08) 0.81 (0.08) 0.817

**Obstruction FEV_1_/FVC < LLN** 1.000 *

**Normal** 83.3 (5) 83.3 (5)

**Mild** 16.7 (1) 16.7 (1)

**Moderate** 0.00 (0) 0.00 (0)

**ACQ Score** 1.000 *

**<0.75** 83.3 (5) 83.3 (5)

**0.75-1.5** 16.7 (1) 16.7 (1)

**>1.5** 0.00 (0) 0.00 (0)

**Bronchodilator Response** 0.258 *

**ΔFEV_1_ (L) < 0.2 L** 66.7 (4) 33.3 (2)

**ΔFEV_1_ (L) > 0.2 L** 33.3 (2) 66.7 (4)

Continuous variables reported as mean (standard deviation) and categorical variable reported as percentage (N). Asthma phenotype (AP) 1 and 2, forced expiratory volume in 1 second (FEV_1_), minimally important difference (MID), forced vital capacity (FVC), and lower limit of normal as determined by ATS/ERS guidelines (LLN), asthma control questionnaire (ACQ).

***** Significance determined by Chi-square analysis with boot-strap approximation of distribution

**Table C. Factor and Vector Fitting for DCA**

**Centroids**

**DCA1 DCA2 R^2^ *P***

**ICS Dose**  0.476 0.879 0.106 0.408 †

**Age** -0.079 0.996 0.336 0.035 †

**Subject** 0.801 0.327 ‡

**Asthma** 0.025 0.608

**N** -0.326 0.209

**Y** 0.082 -0.052

**FP** 0.051 0.391

**N** -0.169 -0.022

**Y** 0.437 0.058

**Group** 0.467 0.037

**Control** -0.326 0.209

**AP1 pre-FP** -1.036 -0.011

**AP2 pre-FP** 0.966 -0.228

**AP1 post-FP** 0.027 0.143

**AP2 post-FP** 0.981 -0.055

**Gender** 0.183 0.088

**Female** -0.435 0.081

**Male** 0.598 -0.112

**BMI** 0.176 0.250

**Normal**  0.408 -0.123

**Obese** -0.652 0.038

**Overweight**  -0.260 0.390

**Obstruction** 0.066 0.589

**Normal** -0.056 0.113

**Mild** -0.161 -0.123

**Moderate** 0.7696 -0.402

All meta-data was fit to deterended correspondence analysis (DCA) as factors except of † ICS dose (0, 100, 500 mg/BID) and Age (range: 19-31 years). ‡ Subjects were fit as factors but individual centroids are not listed. Asthmat Phenotype (AP) 1 and 2, body mass index (BMI), and fluticasone propionate (FP).

|  |  |  |  |  |  |  |  |  |  |  |  |  |  |  |  |  |  |  |  |  |  |  |  |  |  |  |  |  |
| --- | --- | --- | --- | --- | --- | --- | --- | --- | --- | --- | --- | --- | --- | --- | --- | --- | --- | --- | --- | --- | --- | --- | --- | --- | --- | --- | --- | --- |
| 1) Ralstonia solanacearum 2) Ralstonia pickettii 3) Actinomyces odontolyticus 4) Ralstonia sp. 5_7_47FAA 5) Ralstonia sp. 6) Propionibacterium acnes 7) Streptococcus pneumoniae 8) Acidovorax sp. JS42 9) Neisseria meningitidis 10) Streptococcus mitis 11) Acidovorax ebreus 12) Rhodopseudomonas palustris 13) Methylobacterium extorquens 14) Tropheryma whipplei 15) Actinomyces sp. oral taxon 180 16) Rothia mucilaginosa 17) Bradyrhizobium japonicum 18) Cupriavidus metallidurans 19) Burkholderia cenocepacia 20) Propionibacterium freudenreichii 21) Enterococcus faecium 22) Nocardioides sp. JS614 23) Streptococcus salivarius 24) Oribacterium sinus 25) Enterococcus faecalis | 25 | 24 | 23 | 22 | 21 | 20 | 19 | 18 | 17 | 16 | 15 | 14 | 13 | 12 | 11 | 10 | 9 | 8 | 7 | 6 | 5 | 4 | 3 | 2 | 1 |  |  | **Table D. Generalized Linear Model for Top 25 Abundant Taxa Counts using Negative Binominal Distribution** |
|  | 31.1 | 34.1 | 34.3 | 35.8 | 38.6 | 40.1 | 40.9 | 42.5 | 43.1 | 43.5 | 46.5 | 50.0 | 53.9 | 57.0 | 63.6 | 80.6 | 94.5 | 100.4 | 135.5 | 163.7 | 173.4 | 179.1 | 352.9 | 476.7 | 505.7 | **μ** | ***NB*** |  |
|  | 0.2 | 5.1 | 3.3 | 0.7 | 0.3 | 0.5 | 1.7 | 1.5 | 2.0 | 2.2 | 1.8 | 7.5 | 4.4 | 2.1 | 2.1 | 2.3 | 0.9 | 1.6 | 1.4 | 0.9 | 4.9 | 5.3 | 2.1 | 5.0 | 4.1 | **α** |  |  |
|  | 4.7 | 3.6 | 4.3 | 5.0 | 5.0 | 5.1 | 4.7 | 4.6 | 4.4 | 4.4 | 4.6 | 2.7 | 4.9 | 5.4 | 3.5 | 4.8 | 6.1 | 4.2 | 5.8 | 7.1 | 6.3 | 6.4 | 7.3 | 7.8 | 7.9 | **ε** | ***β*** |  |
|  | 0.5 | 0.2 | 1.7 | -0.8 | 0.7 | -1.0 | 0.7 | 1.1 | -0.7 | 1.8 | 1.2 | -1.1 | 1.9 | -0.3 | 4.2 | 0.4 | -1.7 | 4.2 | 0.0 | -1.1 | 1.0 | 1.0 | 1.3 | 1.0 | 1.0 | **C** |  |  |
|  | 1.1 | -1.3 | -0.2 | -1.0 | 1.3 | -0.8 | 1.9 | 2.1 | 0.2 | -2.6 | -2.4 | -2.3 | -0.1 | -0.2 | 0.1 | -0.4 | -0.5 | -0.1 | -0.6 | -0.8 | 2.2 | 2.2 | -2.7 | 2.2 | 2.3 | **AP1** | **Pre** |  |
|  | -0.9 | 1.0 | 0.1 | 0.5 | -0.9 | 0.5 | -0.6 | -0.8 | -0.7 | 0.8 | 1.9 | 1.8 | -1.1 | -0.4 | -1.5 | 2.9 | 1.5 | -1.4 | 2.9 | 0.5 | -0.9 | -0.9 | 2.1 | -0.9 | -0.9 | **AP2** | **Pre** |  |
|  | -0.5 | -2.6 | -2.8 | 0.9 | -0.5 | 1.0 | -0.5 | -0.6 | 2.6 | -1.9 | -1.2 | -2.2 | 1.0 | 1.7 | -1.1 | -3.1 | 0.8 | -0.7 | -1.9 | 1.0 | -0.6 | -0.7 | -1.7 | -0.6 | -0.7 | **AP1** | **Post** |  |
|  | -0.3 | 2.7 | 1.3 | 0.4 | -0.6 | 0.3 | -1.5 | -1.7 | -1.5 | 1.9 | 0.6 | 3.8 | -1.6 | -0.8 | -1.8 | 0.2 | -0.2 | -2.1 | -0.3 | 0.4 | -1.7 | -1.6 | 0.9 | -1.7 | -1.7 | **AP2** | **Post** |  |
|  | 0.1 | 0.6 | 0.5 | 0.2 | 0.1 | 0.2 | 0.3 | 0.3 | 0.4 | 0.4 | 0.4 | 0.7 | 0.6 | 0.4 | 0.4 | 0.4 | 0.3 | 0.3 | 0.3 | 0.2 | 0.6 | 0.6 | 0.4 | 0.6 | 0.5 | **ε** | ***SE*** |  |
|  | 0.2 | 0.9 | 0.8 | 0.5 | 0.3 | 0.4 | 0.6 | 0.6 | 0.7 | 0.7 | 0.6 | 0.9 | 0.8 | 0.7 | 0.7 | 0.7 | 0.5 | 0.6 | 0.6 | 0.5 | 0.9 | 0.9 | 0.7 | 0.9 | 0.8 | **C** |  |  |
|  | 0.2 | 0.9 | 0.8 | 0.4 | 0.3 | 0.4 | 0.6 | 0.6 | 0.7 | 0.7 | 0.7 | 0.9 | 0.8 | 0.7 | 0.7 | 0.7 | 0.5 | 0.6 | 0.6 | 0.5 | 0.9 | 0.9 | 0.7 | 0.9 | 0.8 | **AP1** | **Pre** |  |
|  | 0.2 | 0.8 | 0.8 | 0.4 | 0.3 | 0.4 | 0.6 | 0.6 | 0.6 | 0.7 | 0.6 | 0.9 | 0.8 | 0.6 | 0.7 | 0.7 | 0.4 | 0.6 | 0.5 | 0.4 | 0.8 | 0.9 | 0.6 | 0.8 | 0.8 | **AP2** | **Pre** |  |
|  | 0.2 | 0.9 | 0.8 | 0.4 | 0.3 | 0.4 | 0.6 | 0.6 | 0.6 | 0.7 | 0.6 | 0.9 | 0.8 | 0.7 | 0.7 | 0.7 | 0.5 | 0.6 | 0.6 | 0.5 | 0.9 | 0.9 | 0.7 | 0.9 | 0.8 | **AP1** | **Post** |  |
|  | 0.3 | 0.9 | 0.8 | 0.5 | 0.3 | 0.4 | 0.6 | 0.6 | 0.7 | 0.7 | 0.7 | 1.0 | 0.9 | 0.7 | 0.7 | 0.7 | 0.5 | 0.7 | 0.6 | 0.5 | 0.9 | 0.9 | 0.7 | 0.9 | 0.9 | **AP2** | **Post** |  |

**Table E. Differential Abundance of Top 25 Abundant Taxa Pairwise Comparison AP1/Control**

**L2FC l2fcSE *P* adjusted *P***

**Ralstonia solanacearum**  1.272 1.295 0.326 0.737

**Ralstonia pickettii**  1.198 1.366 0.380 0.758

**Actinomyces odontolyticus** -4.009 1.054 <0.001 0.020

**Ralstonia sp. 5_7_47FAA** 1.176 1.38 0.394 0.767

**Ralstonia sp.** 1.225 1.359 0.367 0.751

**Propionibacterium acnes** 0.213 0.74 0.774 0.904

**Streptococcus pneumoniae** -0.595 0.894 0.506 0.816

**Acidovorax sp. JS42** -4.304 0.956 <0.001 0.004

**Neisseria meningitidis** 1.219 0.764 0.111 0.655

**Streptococcus mitis** -0.75 1.092 0.492 0.811

**Acidovorax ebreus** -4.071 1.054 <0.001 0.020

**Rhodopseudomonas palustris**  0.155 1.052 0.883 0.962

**Methylobacterium extorquens**  -1.989 1.322 NA NA

**Tropheryma whipplei** -1.153 1.498 NA NA

**Actinomyces sp. oral taxon 180** -3.608 1.038 0.001 0.048

**Rothia mucilaginosa** -4.362 1.114 <0.001 0.020

**Bradyrhizobium japonicum** 0.918 1.044 NA NA

**Cupriavidus metallidurans** 1.045 0.935 0.264 0.706

**Burkholderia cenocepacia** 1.223 0.965 0.205 0.681

**Propionibacterium freudenreichii** 0.167 0.628 0.790 0.917

**Enterococcus faecium**  0.528 0.452 0.242 0.685

**Nocardioides sp. JS614**  -0.156 0.715 0.827 0.934

**Streptococcus salivarius**  -1.866 1.232 0.130 0.673

**Oribacterium sinus** -1.487 1.386 NA NA

**Enterococcus faecalis** 0.631 0.382 0.098 0.643

**Table F. Differential Abundance of Top 25 Abundant Taxa Pairwise Comparison AP2/Control**

**L2FC l2fcSE *P* adjusted *P***

**Ralstonia solanacearum** -1.917 1.268 0.131 0.363

**Ralstonia pickettii** -1.96 1.342 0.144 0.378

**Actinomyces odontolyticus** 0.775 1.020 0.447 0.671

**Ralstonia sp. 5_7_47FAA** -1.885 1.357 0.165 0.403

**Ralstonia sp.** -1.885 1.335 0.158 0.401

**Propionibacterium acnes** 1.582 0.714 0.027 0.181

**Streptococcus pneumoniae** 2.909 0.862 0.001 0.035

**Acidovorax sp. JS42** -5.592 0.935 <0.001 <0.001

**Neisseria meningitidis** 3.216 0.736 <0.001 0.002

**Streptococcus mitis** 2.555 1.057 0.016 0.142

**Acidovorax ebreus** -5.685 1.038 <0.001 <0.001

**Rhodopseudomonas palustris**  -0.089 1.023 0.931 0.966

**Methylobacterium extorquens**  -3.021 1.297 NA NA

**Tropheryma whipplei** 2.968 1.468 NA NA

**Actinomyces sp. oral taxon 180** 0.724 0.976 0.458 0.676

**Rothia mucilaginosa** -1.060 1.050 0.313 0.556

**Bradyrhizobium japonicum** 0.021 1.018 NA NA

**Cupriavidus metallidurans** -1.880 0.916 0.040 0.213

**Burkholderia cenocepacia** -1.296 0.943 0.169 0.408

**Propionibacterium freudenreichii** 1.488 0.595 0.012 0.125

**Enterococcus faecium** -1.593 0.450 <0.001 0.023

**Nocardioides sp. JS614** 1.277 0.679 0.060 0.256

**Streptococcus salivarius** -1.583 1.201 0.187 0.429

**Oribacterium sinus** 0.791 1.354 NA NA

**Enterococcus faecalis** -1.360 0.386 <0.001 0.023

**Table G. Differential Abundance of Top 25 Abundant Taxa Pairwise Comparison AP1/AP2**

**L2FC l2fcSE *P* adjusted *P***

**Ralstonia solanacearum** 3.189 1.268 0.012 0.077

**Ralstonia pickettii** 3.158 1.341 0.019 0.100

**Actinomyces odontolyticus** -4.783 1.025 <0.001 0.001

**Ralstonia sp. 5_7_47FAA** 3.061 1.356 0.024 0.115

**Ralstonia sp**. 3.110 1.335 0.020 0.100

**Propionibacterium acnes** -1.370 0.71 0.054 0.185

**Streptococcus pneumoniae** -3.504 0.861 <0.001 0.003

**Acidovorax sp. JS42** 1.288 0.946 0.173 0.366

**Neisseria meningitidis** -1.998 0.72 0.006 0.056

**Streptococcus mitis** -3.305 1.058 0.002 0.030

**Acidovorax ebreus** 1.614 1.049 0.124 0.304

**Rhodopseudomonas palustris** 0.244 1.019 0.811 0.895

**Methylobacterium extorquens** 1.032 1.299 NA NA

**Tropheryma whipplei**  -4.121 1.472 NA NA

**Actinomyces sp. oral taxon 180** -4.333 1.007 <0.001 0.002

**Rothia mucilaginosa** -3.302 1.086 0.002 0.034

**Bradyrhizobium japonicum** 0.897 1.006 NA NA

**Cupriavidus metallidurans** 2.925 0.911 0.001 0.026

**Burkholderia cenocepacia** 2.518 0.937 0.007 0.061

**Propionibacterium freudenreichii** -1.321 0.583 0.023 0.113

**Enterococcus faecium** 2.121 0.441 <0.001 <0.001

**Nocardioides sp. JS614** -1.433 0.672 0.033 0.141

**Streptococcus salivarius**  -0.282 1.204 0.815 0.895

**Oribacterium sinus** -2.278 1.358 NA NA

**Enterococcus faecalis** 1.991 0.373 <0.001 <0.001

**Table H. Differentially Abundant Taxa AP1 pre-FP/AP1 post-FP**

**L2FC l2fcSE *P***

**Actinomyces odontolyticus** 0.973 1.055 0.357

**Actinomyces sp. oral taxon 180** 1.181 1.038 0.255

**Enterococcus faecalis** -1.576 0.369 <0.001

**Enterococcus faecium** -1.802 0.445 <0.001

**Neisseria meningitidis** 1.252 0.744 0.092

**Rothia mucilaginosa** 0.650 1.119 0.562

**Streptococcus pneumoniae** -1.315 0.893 0.141

**Table I. Differentially Abundant Taxa AP2 pre-FP/AP2 post-FP**

**L2FC l2fcSE *P***

**Actinomyces odontolyticus** -1.222 1.057 0.247

**Actinomyces sp. oral taxon 180** -1.310 1.010 0.195

**Enterococcus faecalis** 0.612 0.391 0.117

**Enterococcus faecium**  0.246 0.467 0.597

**Neisseria meningitidis** -1.708 0.748 0.022

**Rothia mucilaginosa** 1.143 1.085 0.292

**Streptococcus pneumoniae** -3.14 0.893 <0.001

**Table J. Differentially Abundant Taxa AP1 post-FP/AP2 post-FP**

**L2FC l2fcSE *P***

**Actinomyces odontolyticus** -2.588 1.085 0.017

**Actinomyces sp. oral taxon 180** -1.842 1.041 0.077

**Enterococcus faecalis** -0.198 0.387 0.609

**Enterococcus faecium** 0.073 0.47 0.877

**Neisseria meningitidis** 0.962 0.771 0.212

**Rothia mucilaginosa** -3.795 1.118 0.001

**Streptococcus pneumoniae** -1.679 0.923 0.069
